# Large-scale spatial population databases in infectious disease research

**DOI:** 10.1186/1476-072X-11-7

**Published:** 2012-03-20

**Authors:** Catherine Linard, Andrew J Tatem

**Affiliations:** 1 Biological Control and Spatial Ecology, Université Libre de Bruxelles, CP 160/12, Avenue FD Roosevelt 50, B-1050 Brussels, Belgium; 2 Fonds National de la Recherche Scientifique (F.R.S.-FNRS), Rue d'Egmont 5, B-1000 Brussels, Belgium; 3 Department of Geography, University of Florida, Gainesville, FL 32611, USA; 4 Emerging Pathogens Institute, University of Florida, Gainesville, FL 32610, USA; 5 Fogarty International Center, National Institutes of Health, Bethesda, MD 20892, USA

**Keywords:** Human population, Global, Infectious diseases, Spatial demography, Health metrics

## Abstract

Modelling studies on the spatial distribution and spread of infectious diseases are becoming increasingly detailed and sophisticated, with global risk mapping and epidemic modelling studies now popular. Yet, in deriving populations at risk of disease estimates, these spatial models must rely on existing global and regional datasets on population distribution, which are often based on outdated and coarse resolution data. Moreover, a variety of different methods have been used to model population distribution at large spatial scales. In this review we describe the main global gridded population datasets that are freely available for health researchers and compare their construction methods, and highlight the uncertainties inherent in these population datasets. We review their application in past studies on disease risk and dynamics, and discuss how the choice of dataset can affect results. Moreover, we highlight how the lack of contemporary, detailed and reliable data on human population distribution in low income countries is proving a barrier to obtaining accurate large-scale estimates of population at risk and constructing reliable models of disease spread, and suggest research directions required to further reduce these barriers.

## Introduction

Mapping and modelling methods used to study the spatial distribution and spread of vector-borne and directly transmitted infectious diseases are becoming increasingly widespread and sophisticated as the field of spatial epidemiology grows. Spatial epidemiology is defined as "the study of spatial variation in disease risk or incidence" [1], and its aims are both to describe and to understand these variations [2], with the ultimate objective being to assist public health decision making. Interactions between pathogens, vectors and hosts, and between these agents and their environment determine spatial variations in disease risk and make the transmission of vector-borne and other infectious diseases an intrinsically spatial process [1,3].

Most studies on infectious disease dynamics are not spatially-explicit, i.e. elements are not explicitly localized in space. Models are typically based on the metapopulation concept, which considers isolated subpopulations subject to colonization and extinction dynamics [4-6]. If the species of interest is a parasite, colonization means infection and a local extinction occurs when the host dies or recovers [5]. This approach is spatially-implicit, as it avoids the use of geographical maps to locate elements. In the majority of non-spatial mathematical models of infectious diseases, the total population is assumed to be constant [7], but population data have been included, for instance, in non-spatial models of HIV [8], pertussis [9], malaria [7], or in global burden of disease calculations [10-16]. However, the spatial nature of infectious diseases, and particularly spatial heterogeneities in transmission and spread, make risk maps and spatially-explicit models of disease incidence valuable tools for understanding disease dynamics and planning public health interventions [1,2,17]. Defining the extent of infectious diseases as a public health burden and their distribution and dynamics in time and space are critical to scoping the financial requirements, for setting a control agenda and for monitoring.

The emergence of spatially-explicit studies in infectious disease research has been supported by improvements in spatial data and tools such as remote sensing and geographical information systems (GIS) [18-23], as well as advances in spatially-explicit modelling methods [17,24]. GIS are commonly used to combine spatial data from different sources, for mapping disease and for performing spatial analyses to identify the causal factors of observed spatial patterns such as cluster detection or landscape fragmentation analyses [20,25]. In addition, the growth in computing, data collection and the centralization of epidemiological data, has lead to an increase in the sophistication and complexity in the mapping and modelling of infectious disease risks.

Among the agents involved in the disease transmission process, human hosts play a crucial role as their density [26], spatial location, demographic characteristics (e.g. age-risk profiles [27-30]) and behaviour [31-33] determine their exposure to infection. Any approach that requires the use of modelled disease rates or dynamics requires reasonable information on the resident population for the time period one is intending to estimate risk. Where risks and spread of diseases are heterogeneous in space, population distributions and counts should ideally be resolved to higher levels of spatial detail than large regional estimates. Accurate and detailed information on population size and distribution are therefore of significant importance for deriving populations at risk and infection movement estimates in spatial epidemiological studies [34]. For many low-income countries of the World, where disease burden is greatest, however, spatially detailed, contemporary census data do not exist. This is especially true for much of Africa, where currently available census data are often over a decade old, and at administrative boundary levels just below national-level [35,36].

Modelling techniques for the spatial reallocation of populations within census units have been developed in an attempt to overcome the difficulties caused by input census data of varying resolutions. National census population data can be represented as continuous gridded population distribution (or count) datasets through the use of spatial interpolation algorithms. Here, we firstly review and compare the methods used in the construction of existing large-scale population datasets, and secondly review applications of these datasets in past studies of disease risk and dynamics.

## Mapping humans

### Spatial demographic data

Our knowledge of human distribution in many areas of the World remains surprisingly poor. A growing interest in the global mapping of human populations emerged in the 1990s [37,38]. Until then, the only information on the spatial distribution of people came from maps showing the location of towns, cities and administrative boundaries on one hand and sparse, inconsistent population data coming from national censuses or demographic surveys on the other [39]. Wright (1936) provided one of the first examples of the combination of demographic and spatial data to build a population density map of the Cape Cod region in the United States [40]. Improvements in demographic and spatial data availability and the development of methods to combine them led to the creation of global population density datasets.

**Figure 1:**
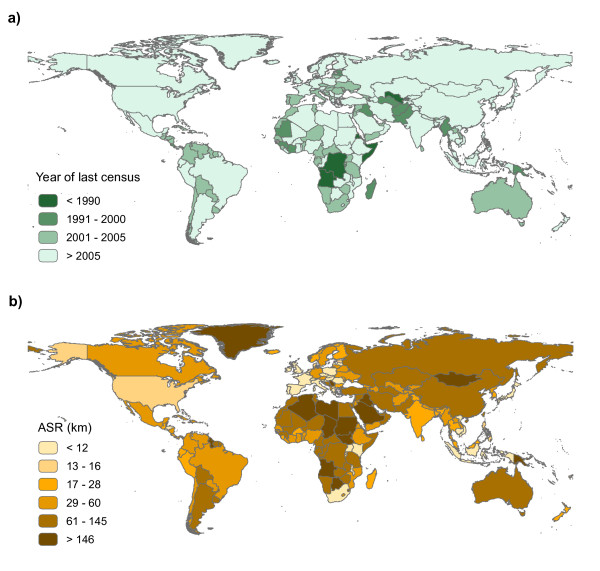
**Spatial and temporal characteristics of available census data**. a) Year of the last national census data available (data source: GeoHive [41]) and b) average spatial resolution (ASR) of census data used in the construction of Gridded Population of the World version 3 (GPW3) and the Global Rural Urban Mapping Project (GRUMP). The ASR measures the effective resolution of administrative units in kilometers. It is calculated as the square root of the land area divided by the number of administrative units [42]. It can be thought of as the "cell size" if all units in a country were square and of equal size.

Demographic data come from different sources: censuses, civil registration systems, governmental or non-governmental administrative data or sample surveys [37]. Civil registration systems provide the most reliable and useful demographic data as they continuously record information on the population of a country, including their spatial distribution. However, up-to-date registration systems only exist in a small number of countries. Instead, censuses are conducted approximately every 10 years by national statistical offices in order to provide consistent and geo-referenced population data. The accuracy and amount of data supplied by national censuses vary considerably from one country to the other. From a temporal point of view, (at the time of writing) the most recent census is more than 25 years old in some sub-Saharan countries such as Angola, Eritrea and the Demographic Republic of Congo [41] (Figure 1a). Large variations also exist in the spatial resolution of available census data, as the ways in which national territories are divided and the administrative level at which population data are collected and summarized vary by country. Figure 1b shows the spatial resolution of census data used in the construction of the Gridded Population of the World version 3 (GPW3) [42] and the Global Rural Urban Mapping Project (GRUMP) [43,44] spatial population databases (both described below).

The link between demographic data and a spatial reference system is essential for geographical analyses. Census data collected at the administrative unit level must be related to an accurate boundary dataset [37]. This has in the past often been neglected, mainly due to a lack of GIS technology, knowledge, resources and methods, as well as computing infrastructure [37,39,42], but efforts are now being made across the world to link census data with digital administrative boundaries.

### Population distribution modelling methods

A variety of methods for converting population count data from irregular administrative units to regular grids have been developed since the 1990s [43,44] and have led to the emergence of differing global gridded population datasets. The quality and accessibility of population and spatial data have been improving, making collaborations between demographers and geographers stronger [39,42,43]. In addition, GIS technologies are becoming increasingly available and accessible and computer power is continuously improving, allowing the processing of larger and more detailed datasets [37,39,42].

**Figure 2:**
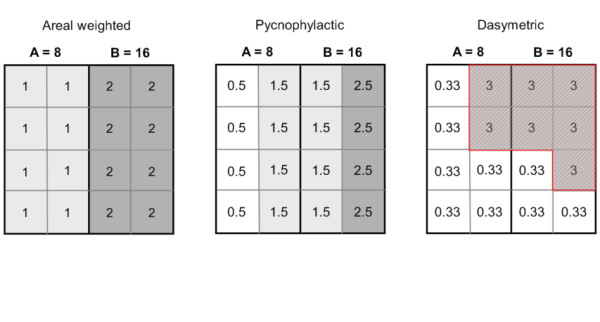
**Schematic illustrations of population distribution modelling methods**. The population of two administrative units A and B (with total population in A = 8 and total population in B = 16) are redistributed according to different population distribution modelling approaches (areal weighted, pycnophylactic and dasymetric). In the dasymetric method, a higher weight was attributed to the red hatched area.

Population distribution modelling methods over large spatial scales rely on redistributing populations within census units to obtain continuous population surfaces, i.e. gridded datasets with a number of inhabitants per grid cell. Different interpolation methods have been typically used to reallocate populations within administrative units (Figure 2):

**Areal weighting **assumes that the population is uniformly distributed within each administrative unit. The population assigned to a grid cell is simply the total population of the administrative unit divided by the number of cells in the administrative unit. Every grid cells of an administrative unit has therefore the same population value [45]. This method was used to construct the Gridded Population of the World (GPW) database, versions 2 and 3 [42,46]

**Pycnophylactic interpolation **starts with the areal weighted method, but smoothes population values using the weighted average of nearest neighbours, while preserving the summation of population data to the original population per areal unit [47]. Pycnophylactic interpolation was used to generate GPW version 1 [48,49].

**Dasymetric modelling **involves using ancillary data - often this may include satellite derived land cover data - to redistribute populations within administrative units [45,50]. Weightings are attributed to the different land cover classes and the population is redistributed accordingly. For example, the Global Rural Urban Mapping Project (GRUMP) uses a similar approach to GPW, but incorporates urban-rural extents and their corresponding populations in the spatial reallocation of census counts [43,44]. The urban-rural extent information is generated by a variety of input data that include census data, online web sources and National Imagery and Mapping Agency (NIMA) database of populated places [46]. The recently produced AfriPop dataset, which covers the African continent at a fine spatial resolution, also used land cover data to redistribute populations [51,52]. Other kinds of ancillary data such as the slope or roads can be used for dasymetric mapping.

More sophisticated modelling approaches - called **smart interpolation **- involve modelling the finescale distribution of populations using a range of satellite and other ancillary data. For example, an accessibility surface developed from road networks and populated places can be used to redistribute people, as was done in the construction of the UNEP database [53-56]. The LandScan dataset is another example of smart interpolation, where various ancillary data such as roads, slope, land cover and nighttime lights are used to determine the probability of population occurrence in cells. Populations are spatially reallocated within each areal unit using modelling approaches based on these probability coefficients [57-59].

Features of each dataset are outlined in Table 1. All of these existing datasets show the spatial distribution of nighttime residential population, except LandScan that maps the 'ambient' population, i.e. the average location of people across time. AfriPop is the only project that also freely provides demographic sub-group gridded datasets, i.e. age composition by 5-years groupings and gender [52]. The most recently updated datasets are LandScan and AfriPop, updated in 2010 and 2011, respectively. However, given its commercial status, the LandScan 2010 dataset is not available in the public domain. LandScan and AfriPop are also the two datasets for which we can expect the most frequent updates in the future. Different levels of transparency in the methodologies are observed. Most of the datasets are fully documented, with methods clearly described and all data sources mentioned (e.g. GPW, GRUMP, UNEP, AfriPop), whereas datasets using more sophisticated interpolation methods are sometimes less transparent. For example, the available documentation of LandScan only enables a general understanding of the methodologies used. These global population distribution datasets that have been created at spatial resolutions of finer than 1 degree have been used in various epidemiological studies, and these are reviewed below.

**Table 1:** Existing gridded global and continental population datasets and their main characteristics.

Code	Dataset	Producer	Method	Level of transparency in data and methodology used	Spatial resolution	Year(s) represented	Updates	Distribution policy	References
GPW	Gridded Population of the World	National Center for Geographic Information and Analysis (NCGIA), University of California; Center for International Earth Science Information Network (CIESIN), Columbia university	GPW1: pycnophylactic; GPW2 and GPW3: areal-weighted	High	2.5 arcminutes (~5 km)	1990, 1995, 2000, 2005, 2010^1^, 2015^1^	1995,2000,2004	Open-access	[42,46,48,49]

GRUMP	Global Rural Urban Mapping Project	Center for International Earth Science Information Network (CIESIN), Columbia university; International Food Policy Research Institute; The World Bank; Centro Internacional de Agricultura Tropical	Dasymetric	High	30 arcseconds (~1 km)	1990, 1995, 2000	2000, 2004	Open-access	[44]

LandScan	LandScan Global Population database	Oak Ridge National Laboratory	Smart	Low	30 arcseconds (~1 km)	year of release	1998; yearly from 2000 to 2010	Commercial	[57-59]

UNEP	UN Environment Programme global population datasets	United Nations Environment Programme/Global Resource Information Database (UNEP/GRID), Sioux Falls	Smart	High	2.5 arcminutes (~5 km)	2000	1996, 2004	Open-access	[53-55]

AfriPop	AfriPop population dataset for Africa	AfriPop project: University of Oxford, University of Florida and Université Libre de Bruxelles	Dasymetric	High	3 arcseconds (~100 m)	2010	2011	Open-access	[51,52]

^1 ^Based on extrapolations of older datasets using UN growth rates

### Uncertainty and error

Given the different input data and the different modelling methods used, the existing gridded population datasets described above clearly differ. Different sources of error and uncertainty are associated with these population datasets, which generally arise from (i) the input data, (ii) temporal projections and (iii) the modelling procedure used.

**Figure 3:**
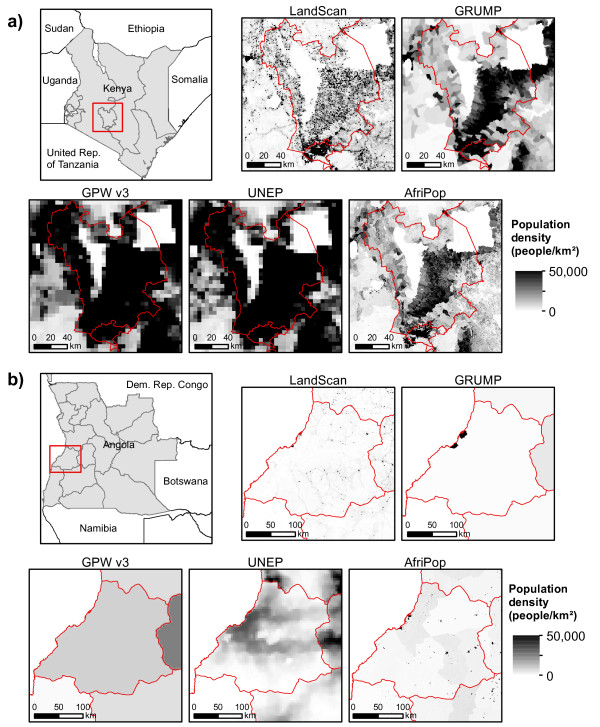
**Selected examples of existing global and continental population datasets**. LandScan 2008, GRUMP beta version, GPW3, UNEP Africa and AfriPop for a) a region in Kenya where census data is very detailed and b) a region of Angola where census data is coarse.

Uncertainties associated with input data, such as census data, can be important, especially in low income regions where misreporting errors may be frequent [60,61]. In addition to errors in population counts, inaccuracies in the spatial positioning of administrative unit boundaries can lead to population mapping uncertainties. Population movements also make counts not entirely representative of the long-term residential population. However, censuses are often the only consistent and exhaustive population databases available in countries where registration systems do not exist, and quantifying such uncertainties remains difficult. Figure 3 shows population distribution as mapped by existing global population datasets (LandScan 2008, GRUMP beta, GPW3, AfriPop and UNEP Africa) for a region of Kenya, where census data are available at a high administrative unit level, and a region of Angola, where the spatial resolution of census data is coarse. This figure highlights how the differing approaches to the spatial interpolation of census data produce very different spatial configurations of population distribution when census data are aggregated in large administrative units, as in the case of Angola. Recent studies used Kenya data at different administrative levels to show quantitatively that population map accuracies significantly improve with finer resolution input census data [35,62,63]. With fine resolution census data, populations are already distributed in small spatial units, so that the margin of possible errors due to the population distribution modelling is reduced, relative to the output resolution of the modelled surfaces. These studies demonstrate that obtaining as fine a spatial resolution of census data as possible must be the priority starting point in population distribution modelling [35,62,63]. This calls for regular updating of input census data, as and when it becomes available. The cost of population censuses is however large and most countries undertake full censuses only once per decade. Therefore, models also help provide estimates in the intervening years. Given the positional uncertainties that may be associated with detailed boundaries, some dataset producers (e.g. LandScan) have prioritised smart modelling methods over obtaining fine resolution census data. Deciding upon which solution produces the more accurate results here is difficult. However, if the methodologies used to construct the fine resolution census data boundaries are well documented and known to match accurately with census enumeration units, then the use of detailed census data likely produces consistently more accurate results in mapping [35].

To adjust population counts to one target year, either inter-censal growth rates or national-level growth rates - often from the United Nations [64] - are used. These national estimates are derived from fertility, mortality and international migration numbers, a method that is inevitably associated with uncertainties [65]. In addition, growth rates can vary substantially within countries, introducing uncertainties when using national-level estimates, and are dependent upon the urban-rural definition used when using urban and rural growth rates.

Modelling approaches using ancillary datasets only increase population distribution model accuracies over the simple gridding (areal weighting) of census data if the ancillary data is more detailed and complete spatially than the input census data, and can be detrimental to modelling accuracies otherwise [35,62]. The extent to which ancillary data can improve population model accuracies depends on the resolution of census data, and decreases when the resolution of census data becomes finer.

The validation of large-scale population distribution datasets is problematic as no independent source exists that could serve this purpose globally. Map accuracies can be tested in target regions where reference data are available at a finer spatial resolution than the map produced [57,62,66,67]. Recent studies have shown that using certain methods of downscaling increases consistently the mapping accuracy over simple areal weighting of administrative unit census data [35,63]. Until now, validation efforts at the global level have been limited to comparing results with population totals reported by the UN (in the case of GPW and GRUMP) [39]. Another way to evaluate the accuracy of population datasets is to use geospatial metrics to compare spatial datasets [68]. These metrics quantify differences in the spatial structure of datasets and analyse properties such as spatial correlations within and between datasets [68,69]. The accuracy of urban extent datasets can be more easily assessed using expert opinion [70] or a set of independent test sites derived from medium or high resolution remote sensing imagery [71,72]. In any case, a future priority should be to design methods for the incorporation of uncertainty explicitly.

## Application of global population distribution data in studies of disease risk and dynamics

Gridded population data have been commonly used to estimate populations at risk of infectious disease and to simulate disease dynamics. These raster datasets have the advantage of being global and consistent in terms of spatial resolution and are particularly useful for population at risk (PAR) assessments and infectious disease risk mapping and modelling. In this section, we will first review studies that have used gridded population datasets as input data for spatial transmission models. Secondly, studies that used existing gridded population data to calculate infectious disease health metrics, such as populations at risk, will be covered. An overview of the literature cited is available in Table 2.

**Table 2:** Infectious disease-related studies that have utilized large-scale spatial population databases (adapted from [34])

Disease	Application	Population map used [Reference]
Malaria	Populations at risk	GPW [108,122,123,132,134,135,138], Landscan [109,133], UNEP [107], GRUMP [110,113-115,124,127,128,139]
	Clinical cases	GPW [111], GRUMP [112]
	
	Intervention coverage	GRUMP [126]
	
	Funding coverage	GRUMP [129]
	
	Risk mapping	GPW [81,88,90], UNEP [82], GRUMP [110]
	
	Infection movements	GRUMP [128], GRUMP and AfriPop [101]
	
	Urbanization effects	GPW [78], GRUMP [36]

Helminths	Populations at risk	GPW [118,122,123,140,141], GRUMP [142,143], UNEP [117,119]
	
	Risk mapping	Landscan [89]

Influenza	Epidemic modelling	GPW [94,95,99,100], Landscan [92,93,96], GRUMP [91]
	
	Risk mapping	GRUMP [85], Landscan [79,83]

Yellow fever	Populations at risk	GRUMP [80]

Dengue	Populations at risk	GRUMP [80]
	
	Risk mapping	UNEP [121], Landscan [86,87]

Trypanosomiasis	Populations at risk	Landscan [120]
	
	Risk mapping	UNEP [77]

Bovine TB	Risk mapping	Landscan [84,144], GPW [144]

HIV	Prevalence analyses	Landscan [76]

Leprosy	Risk mapping	GPW [75]

Poliovirus	Incidence analyses	GPW [74]

General	Trends in emerging diseases	GPW [73]
	
	Health of schoolchildren	UNEP [145]

### Input data for spatial transmission modelling

Population density and growth are significant drivers for the emergence of different categories of infectious diseases [73]. Jones and colleagues (2008) examined the relationship between the spatial distribution of emerging infectious disease events of different kinds - including vector-borne and non-vector-borne - and population data from GPW3 [73]. For directly-transmitted diseases, the spatial distribution of people is determinant as it controls person-to-person contacts and therefore the spread of the disease. Gridded population data have been used in spatio-temporal models that simulate contacts between infectious and susceptible people and the spatial spread of the disease [74-76]. Even if less obvious, the link between indirectly-transmitted diseases - i.e. infections that require an external agent for transmission to occur, such as a vector, an animal host or the environment - and population density can also be determinant. The effect however varies according to the disease considered, mainly because population size and distribution can modify the habitat of disease vectors or hosts and hence increase or decrease disease incidence. For example, trypanosomiasis is expected to decline in large parts of Africa because of the growth in human population and the expansion of agriculture at the expense of tsetse fly habitat [77]. Urbanization in Africa is also expected to reduce malaria transmission risk [78]. Other transmitting agents can live in urbanized or highly populated places, making the disease transmission risk higher in these areas, such as poultry responsible for avian influenza [79].

Gridded population datasets have been widely used to examine the relationships between infectious disease incidence and population size and distribution. Globally, population density has an opposed impact on dengue and yellow fever: vector preferences mean that dengue risk is higher in highly populated urban areas, whereas yellow fever risk is higher in rural areas [80]. Several authors included gridded population data as a risk factor in disease mapping and modelling [79,81-85]. When the objective is to study the impact of factors other than population (e.g. climate, ecological or socio-economic variables) on disease transmission, gridded population data can be used as an offset variable in statistical models, i.e. to control for population count differences by analysing rates instead of absolute values [86,87].

Besides spatial models that simply study the statistical association between disease risk or incidence and population density in order to map disease risk, more sophisticated spatially-explicit models have been developed to study the spatial diffusion of infectious diseases. Several of such spatially-explicit models have successfully used gridded population datasets as input data, for example for creating risk maps [88,89], for the calculation of a global malaria transmission stability index [90], or to study the potential economic impacts of avian influenza in Nigeria [91]. All of these models were developed at the grid cell level, making gridded population datasets particularly useful. Gridded population data have also been used to develop agent-based simulation models at the regional level [92,93] and at the global level [94]. Whatever the spatial approach for modelling - patch, distance, group or network - population data are essential, as these models generally require the generation of a virtual society with an appropriate distribution of people. Population distribution datasets have been used to randomly distribute households in study areas according to local population densities [92-94]. In these models, gridded population data provide valuable input datasets mainly because of their wide coverage, consistent spatial detail and availability in the public domain. Moreover, most spatially-explicit models are grid cell-based, meaning that the gridded population datasets are ready to use without any further processing.

Human interactions and movements are crucial for disease spread. However, the complexity of human mobility and its multiscale nature make comprehensive data on movements difficult to obtain [95]. A recurrent issue emerging from large-scale modelling of infectious diseases is therefore the mobility patterns and the spatial details required for transport network data [95]. Gridded population data provide a useful baseline for developing mobility networks. Several authors have combined gridded population data with transport networks to simulate the large-scale spread of infectious diseases [95-100]. In low-income countries, data on population flows are rare and a recent study on the risk of introducing malaria to areas targeted for elimination used a database of bilateral migrant stocks and the GRUMP dataset to evaluate international population movements [101]. Even if population datasets help in the construction of large-scale mobility databases, significant further work is required to fill the data gaps.

### Endemic disease health metrics

Population distribution datasets constitute an essential denominator required for many infectious disease studies. It is well known that disease transmission is focal and heterogeneous [102-105], partially due to the clustered nature of human population distribution. As the precision and detail of disease risk mapping improves, spatial population datasets that capture these patterns are therefore required if the sizes of populations at risk (PAR) are to be more accurately quantified.

PAR of some infectious diseases have been estimated based on gridded population datasets, principally malaria [78,106-115], helminth infections [116-119], human African trypanosomiasis [120] and dengue and yellow fevers [80,121]. Typically, disease risk maps are spatially overlaid onto population distribution datasets to quantify the number of people residing in specific risk zones or classes, and thus derive PAR numbers. This commonly used method of combining population and prevalence data to assess PAR was also used to study co-infection of diseases that show a clear geographic overlap, such as malaria and helminth infections [122,123]. This method is simple to implement and provides a useful assessment of PAR. However, PAR assessments generally use the different existing population datasets interchangeably to provide such estimates. Moreover, uncertainties inherent in the population datasets are rarely acknowledged in such calculations. As already described above, existing gridded population datasets clearly differ, especially where census population data are spatially coarse, and both input-based and process-based uncertainties contribute to great variations in mapping precision (Figure 3). As a consequence, large variations in PAR estimates can result from the choice of population dataset, particularly in low-income countries where census data are often spatially and temporally poor [34]. Specific estimates for pregnant women and children have been also derived from population distribution datasets, by combining gridded population data with age, sex and fertility data from the United Nations [124,125]. However, given that the demographic composition of populations varies substantially within countries, using such national-level demographic estimates introduces additional uncertainties, as already mentioned before.

The combination of endemic disease risk maps with human population counts in the 'at risk' regions presents opportunities for designing the targeting of interventions such as resource allocation, vaccine campaigns or epidemic prevention measures to regions where they will have most cost-effective or burden-reducing impact [80,108,111]. For example, PAR estimates of malaria enabled the derivation of intervention coverage estimates [126], the intervention costs for reducing the malaria burden [127], malaria elimination feasibility [128], and the actual funding coverage [129]. The use of gridded population datasets in these studies facilitated more precise estimates of PAR than estimates based on aggregated population data by administrative units. An accurate assessment of 'at risk' populations also presents opportunities for designing disease surveillance and early warning systems for epidemics in the populations [130,131].

The size of PAR is expected to vary in the future as a response to environmental and demographic changes. Some authors have attempted to assess the future PAR of malaria according to different scenarios [132-135]. While climate factors are predicted to cause limited changes in PAR of malaria for the year 2050 [134] and even a reduction in the size of PAR in Africa in the coming decades [133], demographic changes could significantly increase PAR. Applying forecasted population growth rates to gridded population data enables the derivation of estimates of future global population distribution [132,135]. The combination of such projected data based on GPW2 with climate scenarios showed that population growth likely will have a larger effect than climate change on future global PAR of malaria estimates [135]. Moroever, a study examining the combined effects of climate, population and urbanisation changes confirmed the likely dominant effect of population growth in the increasing size of malaria PAR estimates in Africa [132]. Dengue fever risk in the future was also estimated based on population and climate projections for 2055 and 2085 [121].

## Conclusions

Spatial methods and tools are now widely used in infectious disease research and have led to significant advances in our understanding of disease dynamics, surveillance and control [1,2,17,18]. Population distribution datasets are becoming increasingly important inputs to these models. During the past decade, a number of advances have been made in GIS technologies that have allowed demographers and GIS specialists to begin to map the spatial distribution of human populations globally at an unprecedented level of detail. We have shown in this paper how useful large-scale gridded population datasets are for the calculation of PAR of infectious diseases and for disease risk mapping and modelling. Gridded population datasets allow the user to select geographic boundaries of interest independently from administrative boundaries. Population datasets capture spatial heterogeneities observed in disease transmission risks, making PAR calculations significantly more accurate than can be reached with aggregated population data.

Nevertheless, a number of issues and challenges remain, that, if resolved, would permit a more refined analysis of the spatial distribution of human population around the globe, and reduced uncertainty resulting from their use in epidemiological studies. Population distribution modelling methods have raised several issues and challenges such as the lack of comparability of statistical data from different countries and sources [43], the lack of standard definitions for what constitutes an urban area [136], the need for extensive source and metadata information, and the difficulties in validating the existing population datasets or measuring which existing dataset is the most accurate. The construction of contemporary, well-validated and well-documented spatial demographic datasets should be a priority in order to reduce uncertainties in spatial epidemiological studies [34]. In the absence of a more institutionalized mechanism to generate updated and freely available population datasets, data sharing should be encouraged between projects. Each dataset is for instance built upon similar population data linked to administrative boundaries and a standardized database framework that would encourage sharing of new and improved datasets between projects would greatly facilitate the data production and benefit the users.

Several extensions of population datasets would be particularly useful for infectious disease research (as well as other health related fields such as disaster risk management or conservation), for policy and planning. The most useful one would be to improve information on population attributes of interest. The disease impact in terms of morbidity, mortality, and speed of spread varies substantially with demographic profiles, so that identifying the most exposed or affected populations becomes a key aspect of planning and targeting interventions. It is not feasible for global population databases to generate on-demand maps for each variable of interest, nevertheless the potential to leverage current freely available population databases appears large, as was recently discussed in Tatem et al. [137]. These authors proposed a strategy for building an open-access database of spatial demographic data that is tailored to epidemiological applications.

Over the next few years, improvements in population distribution modelling methods and infectious disease distribution mapping will allow further refinements in PAR estimation and intervention targeting. Continued efforts to resolve the remaining challenges in accurate spatial population datasets construction will be required to obtain the full benefits from these potentially powerful methodologies.

## Competing interests

The authors declare that they have no competing interests.

## Authors' contributions

CL conducted the core literature review and drafted the manuscript. AJT helped to draft the manuscript and revised it critically. All authors read and approved the final manuscript.
